# Second Look After Retromuscular Repair With the Combination of Absorbable and Permanent Meshes

**DOI:** 10.3389/fsurg.2020.611308

**Published:** 2021-01-08

**Authors:** Alvaro Robin Valle de Lersundi, Joaquín Munoz-Rodriguez, Javier Lopez-Monclus, Luis Alberto Blazquez Hernando, Carlos San Miguel, Ana Minaya, Marina Perez-Flecha, Miguel Angel Garcia-Urena

**Affiliations:** ^1^Hospital Universitario del Henares, Madrid, Spain; ^2^Universidad Francisco de Vitoria, Madrid, Spain; ^3^Hospital Universitario Puerta de Hierro Majadahonda, Madrid, Spain; ^4^Ramón y Cajal University Hospital, Madrid, Spain

**Keywords:** second look, absorbable mesh, polypropylene mesh, mesh integration, posterior component separation, transversus abdominis release

## Abstract

**Objective:** The aim of this study is to describe the macroscopic features and histologic details observed after retromuscular abdominal wall reconstruction with the combination of an absorbable mesh and a permanent mesh.

**Methods:** We have considered all patients that underwent abdominal wall reconstruction (AWR) with the combination of two meshes that required to be reoperated for any reason. Data was extracted from a prospective multicenter study from 2012 to 2019. Macroscopic evaluation of parietal adhesions and histological analysis were carried out in this group of patients.

**Results:** Among 466 patients with AWR, we identified 26 patients that underwent a reoperation after abdominal wall reconstruction using absorbable and permanent mesh. In eight patients, the reoperation was related to abdominal wall issues: four patients were reoperated due to recurrence, three patients required an operation for chronic mesh infection and one patient for symptomatic bulging. A miscellanea of pathologies was the cause for reoperation in 18 patients. During the second surgical procedures made after a minimum of 3 months follow-up, a fibrous tissue between the permanent mesh covering and protecting the peritoneum was identified. This fibrous tissue facilitated blunt dissection between the permanent material and the peritoneum. Samples of this tissue were obtained for histological examination. No case of severe adhesions to the abdominal wall was seen. In four cases, the reoperation could be carried out laparoscopically with minimal adhesions from the previous procedure.

**Conclusions:** The reoperations performed after the combination of absorbable and permanent meshes have shown that the absorbable mesh acts as a protective barrier and is replaced by a fibrous layer rich in collagen. In the cases requiring new hernia repair, the layer between peritoneum and permanent mesh could be dissected without special difficulty. Few intraperitoneal adhesions to the abdominal wall were observed, mainly filmy, easy to detach, facilitating reoperations.

## Introduction

Treatment of complex incisional hernias of the anterolateral abdominal wall is a surgical challenge. These defects are usually repaired with non-absorbable materials to minimize hernia recurrence. There are several surgical planes to insert meshes: intraperitoneal (inlay), preperitoneal, retro-muscular (sublay) or supra-aponeurotic (onlay) (1). Onlay mesh placement presents more postoperative complications but comparable recurrence rate to sublay techniques (2). Synthetic meshes placed intra-peritoneally can cause dense adhesions, bowel injuries, mesh migrations and mesh erosion into abdominal contents, although the risk of enterocutaneous fistula formation remains low. Unlike the aforementioned planes, retrorectal mesh placement might offer advantages especially when dealing with complex hernia repair. Posterior components separation technique (PCS) as described by Novitsky and Rosen has shown beneficial effects (3). In fact, transversus abdominis release (TAR) is nowadays one of the most effective approaches for complex abdominal wall reconstruction (4). Once the plane between the peritoneum/transversalis fascia and the muscular plane has been created, permanent meshes are commonly used. However, performing retromuscular abdominal wall reconstruction (AWR) is a challenging procedure that may lead to serious complications. Bowell injuries, adhesions to intra-abdominal contents, internal hernias through openings on the peritoneum and posterior rectus sheaths, postoperative pain due to transparietal fixations and mesh wrinkling or migration due to permanent mesh structure are the most common. Those reasons guided us to develop a strategy using two types of meshes in the same retro-muscular plane, an absorbable mesh (AM) along with a non-absorbable permanent mesh (PM) (5, 6). There are also concerns about reoperations after retromuscular mesh placement in terms of early reoperations due to complications or late operations for recurrences or another surgical causes. The aim of this study is to report the macroscopic evaluation and histological features observed among 26 patients that underwent a reoperation after retromuscular abdominal wall reconstruction with AM and PM for complex incisional hernia repair.

## Methods

From a prospectively maintained database of complex abdominal wall repair in two hospitals, we identified patients who were reoperated for any reason, between April 2012 and December 2019. The two hospitals involved in the study are recognized referral centers for AWR. All patients underwent an AWR using the combination of AM and PM, as previously described (5). The AM used is made of polyglycolic acid and trimethylene carbonate (GORE^®^ BIO-A^®^ Tissue Reinforcement, WL Gore & Associates, Inc. Flagstaff, AZ, USA). We used two types of macroporous PM: a 26 × 36 cm, 60 g/m^2^ polypropylene mesh (Optilene mesh, B. Braun, Melsungen, Hessen. Germany) or a 50 × 50 48 g/m^2^ polypropylene mesh (Bulevb^®^, Dipro Medical Devices SRL, Torino, Italy).

All reoperations except one were performed in the same centers as the index procedures. Demographics and patient characteristics are summarized in Table 1. In those cases, in which the adhesions to the abdominal wall could be re-explored, we used the adhesion tenacity score to assess intraperitoneal attachments to implanted meshes (7, 8).

**Table 1:** Demographics and characteristics of patients.

Male Female	11 (42.3%) 15 (57.7%)
Age, mean ± DS	63.25 ± 8.55
BMI, mean ± DS	31.39 ± 7.12
Obesity (BMI >30)	13 (50%)
Comorbidities Smoking Anticoagulation Diabetes Immunosuppression Hypertension Neoplasia Cardiac Disease Renal Disease Liver Disease	2 (7.6%) 2 (7.6%) 4 (15.3%) 5 (9.2%) 13 (50%) 9 (34.6%) 2 (7.6%) 2 (7.6%) 5 (19.2%)
CeDAR; median (min–max)	28.30 (11–55)
ASA I II III IV	2 (7.6%) 14 (53.8%) 10 (38.4%) 0 (0%)
Prior history of hernias	5 (19.2%)
Number of previous incisional hernia repairs, median (min–max)	1 (0–12)
Etiology of main IH Digestive tube Liver-pancreatic Urology Abdominal wall Gynecology and obstetrics	13 (50%) 5 (19.2%) 5 (19.2%) 2 (7.6%) 1 (3.8%)

*BMI, Body mass index; CeDAR, Carolinas Equation for Determining Associated Risks; ASA, American Society of Anesthesiologists; IH, Incisional hernia*.

We obtained samples of tissue in six reoperated patients, that were stained for microscopic analysis with hematoxylin-eosin stain, Masson's trichrome and Picrosirius red stain to asses tissue integration of the meshes (9).

We have adhered to the STROBE Statement recommendations to draft this article (10). All patients provided informed consent to be included in prospective studies prior to surgery. We obtained Institutional Review Board approval.

### Statistics

We have used Statistical Package for the Social Sciences (SPSS) program (version 19.0 for Windows) to describe variables and for statistical analysis. Quantitative variables were expressed as mean or median and standard deviation or quartiles, and categorical variables as absolute numbers and percentages.

## Results

We identified 26 patients that had previously undergone AWR with PCS and reconstruction with the combination of AM and PM repair and required ensuing reoperation. Patient demographics and characteristics are shown in Tables 1, 2. Operative details of previous AWR are summarized in Table 3. It is worth noting that among the 26 selected patients, most of the patients underwent a posterior component separation technique as previously described (6, 11, 12). In three cases, lateral retromuscular preperitoneal approach was performed and, in a patient with parastomal hernia, modified Pauli technique was achieved (13).

**Table 2:** Characteristics of incisional hernias.

EHS classification of IH Miline M1–M3 M1-M4 M1-M5 M2-M5 M3–M5 Lateral L1 L3 L4 Midline + Lateral Parastomal Parastomal grade III Parastomal grade IV	8 (30.7%) 1 (3.8%) 2 (7.6%) 2 (7.6%) 1 (3.8%) 2 (7.6%) 7 (26.9%) 2 (7.6%) 2 (7.6%) 3 (11.5%) 6 (23.1%) 5 (19.2%) 2 (7.6%) 3 (11.5%)
Width of main IH (EHS) W1 (<4 cm) W2 (4–10 cm) W3 (>10 cm)	0 (0%) 9 (34.6%) 17 (65.3%)
Maximum horizontal size cm of main IH; median (min–max)	11.93 (5–25)
Maximum vertical size cm of main IH; median (min–max)	11.8 (6–24)
Slater's classification of main IH Grade 1 Grade 2 Grade 3	0 (0%) 15 (57.7%) 11 (42.3%)
VHWG classification of main IH Grade 1 Grade 2 Grade 3 Grade 4	4 (15.3%) 14 (53.8%) 8 (30.7%) 0 (0%)
VHSS classification of main IH Grade 1 Grade 2 Grade 3	3 (11.5%) 17 (65.3%) 6 (23.1%)

*EHS, European Hernia Society; VHWG, Ventral Hernia Working Group hernia classification; VHSS, Ventral Hernia Staging System classification*.

**Table 3:** Operative data of abdominal wall reconstruction.

Type of surgery Elective Urgent	25 (96.1%) 1 (3.8%)
Wound classification Clean Clean-contaminated Contaminated Dirty	16 (61.5%) 5 (19.2%) 5 (19.2%) 0 (0%)
Surgical technique Midline Posterior component separation Lateral Lateral Retromuscular preperitoneal Reverse PCS Parastomal PCS + keyhole parastomal repair Modified Pauli parastomal repair	14 (53.8%) 3 (7.6%) 4 (15.4%) 4 (15.4%) 1 (3.8%)
Associated surgery to the IH repair Intestinal resection Closure of bowel opening Another abdominal surgery	2 (7.6%) 2 (7.6%) 5 (19.2%)
Operative time (min), mean (range)	250 (168–300)

*PCS, Posterior component separation*.

In eight cases, the reoperation was centered on the abdominal wall as four patients presented hernia recurrence, three patients chronic mesh infection, and one patient abdominal postoperative symptomatic bulging. The remaining 18 patients were reoperated for a variety of diseases that included intestinal leak, intestinal obstruction, bariatric surgery, oncological disease, symptomatic cholelithiasis and hiatal hernia. Operative data regarding reoperations are summarized in Table 4. Among those five that required new abdominal wall surgery, we performed a retromuscular redo repair in four cases: two patients underwent retromuscular modified Pauli repair (13), one patient retromuscular reverse TAR (12) and one patient a redo Rives-Stoppa procedure. We accomplished anterior components separation technique in one patient.

**Table 4:** Operative data regarding reoperations.

**Etiology of second intervention**		***N* (%)**	**Type**	**Timing**	**Adhesions**	**Biopsy**	**Surgical procedure**
**Abdominal wall**		**8 (30.7%)**					
Chronic mesh infection		3 (11.5%)					
	MRSA		Elective	8 months	^†^		Partial mesh removal
	MRSA		Elective	6 months	^†^		Partial mesh removal + VAC therapy
	*Staphylococcus aureus*		Elective	6 months	^†^		Partial mesh removal
Recurrence (EHS class)		4 (15.3%)					
	M1–M3 W2		Elective	34 months	1	Yes	Retromuscular Rives
	Parastomal grade II		Elective	12 months	0	Yes	Retromuscular modified Pauli repair
	Parastomal grade II		Elective	15 months	1	Yes	Retromuscular modified Pauli repair
	M1–3 W2		Elective	25 months	1		Anterior component separation
Bulging	L4	1 (3.8%)	Elective	38 months	0	Yes	Retromuscular reverse TAR
**Intraabdominal surgery**		**18 (69.3)**					
Oncological disease		4 (15.3%)					
	Colon carcinoma		Elective	40 months	0		Right colectomy
	Cholangiocarcinoma recurrence		Elective	16 months	1		IV segment hepatic resection
	Pancreatic adenocarcinoma		Elective	59 months	2		Subtotal pancreatectomy
	Colon carcinoma		Elective	38 months	0		Right colectomy
Cholelithiasis		2 (7.6%)					
	Cholecystectomy		Elective	24 months	1		Open cholecystectomy
	Cholecystectomy		Elective	14 months	1		Laparoscopic cholecystectomy
Intestinal obstruction		3 (11.5%)					
	Adhesion in small bowel		Urgent	7 months	1		Laparoscopic adhesiolysis small bowel
	Adhesion to pelvis^*^		Emergency	24 months	1	Yes	Pelvic adhesiolysis
	Adhesion to pelvis^*^		Urgent	4 months	0	Yes	Ileocecal resection
Obesity		1 (3.8%)					
	Obesity surgery		Elective	31 months	2		Laparoscopic sleeve gastrectomy
Peritonitis		6 (23.0%)					
	Inadvertent enterotomy		Emergency	2 days	^‡^		Closure of small bowel
	Inadvertent enterotomy		Emergency	10 days	2		Anastomosis
	Inadvertent enterotomy		Emergency	1 day	^‡^		Closure of small bowel
	Inadvertent enterotomy		Emergency	1 day	^‡^		Closure of small bowel
	Colon erosion on stoma site		Emergency	21 days	^‡^		New stoma site
	Inadvertent enterotomy		Emergency	2 days	^‡^		Closure of small bowel
Hiatal hernia		2 (7.6%)					
	Hiatal hernia		Elective	1.5 months	1		Laparoscopic Nissen
	Gastric volvulus		Urgent	3 days	1		Esophagectomy

*MRSA, Methicilin resistant Staphylococcus aureus; VAC, vacuum assisted closure*.

* *Both patients had oncologic abdominoperineal resection with total mesorectum excision as first operation*.

† *Adhesions were not explored*.

‡ *Reoperated too early to evaluate adhesions*.

*Adhesion Tenacity score (8)*.

*0: No adhesion*.

*1: Filmy adhesion: viscera/omentum not attached to mesh, disrupted manually*.

*2: Dense adhesion: viscera/omentum attached to mesh requiring blunt dissection to separate viscera/omentum from mesh*.

*3: Dense adhesion: viscera/omentum attached to mesh requiring sharp dissection to separate viscera/omentum from mesh*.

*4: Dense adhesion: viscera/omentum entwined in the mesh requiring sharp dissection to separate mesh from abdominal wall, leaving mesh attached to viscera/omentum*.

According to the adhesion tenacity classification used: 10 patients presented filmy adhesions that were manually disrupted (grade 1), three dense adhesions that required blunt dissection (grade 2) and five presented no adhesions (grade 0). It is particularly important to emphasize that none of the patients presented grade 3 or 4 adhesions that required sharp dissection or leaving fragments of mesh attached to the viscera. From this analysis, the three patients with chronic infection were not included as the abdominal cavity were not re-explored. Those patients reoperated in the immediate postoperative period (<7 days) were also excluded.

**Figure 1:**
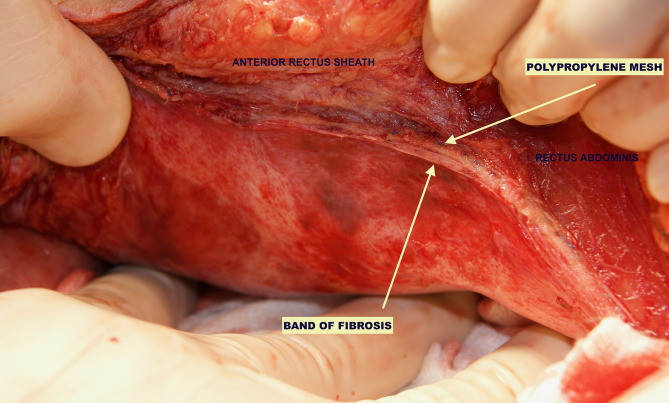
Case of reoperation at 4 months for intestinal obstruction to pelvic floor. No intra-abdominal adhesions to the abdominal wall were seen. The permanent mesh could not be discerned through the peritoneum. A layer of fibrosis between the peritoneum and the permanent mesh is observed.

**Figure 2:**
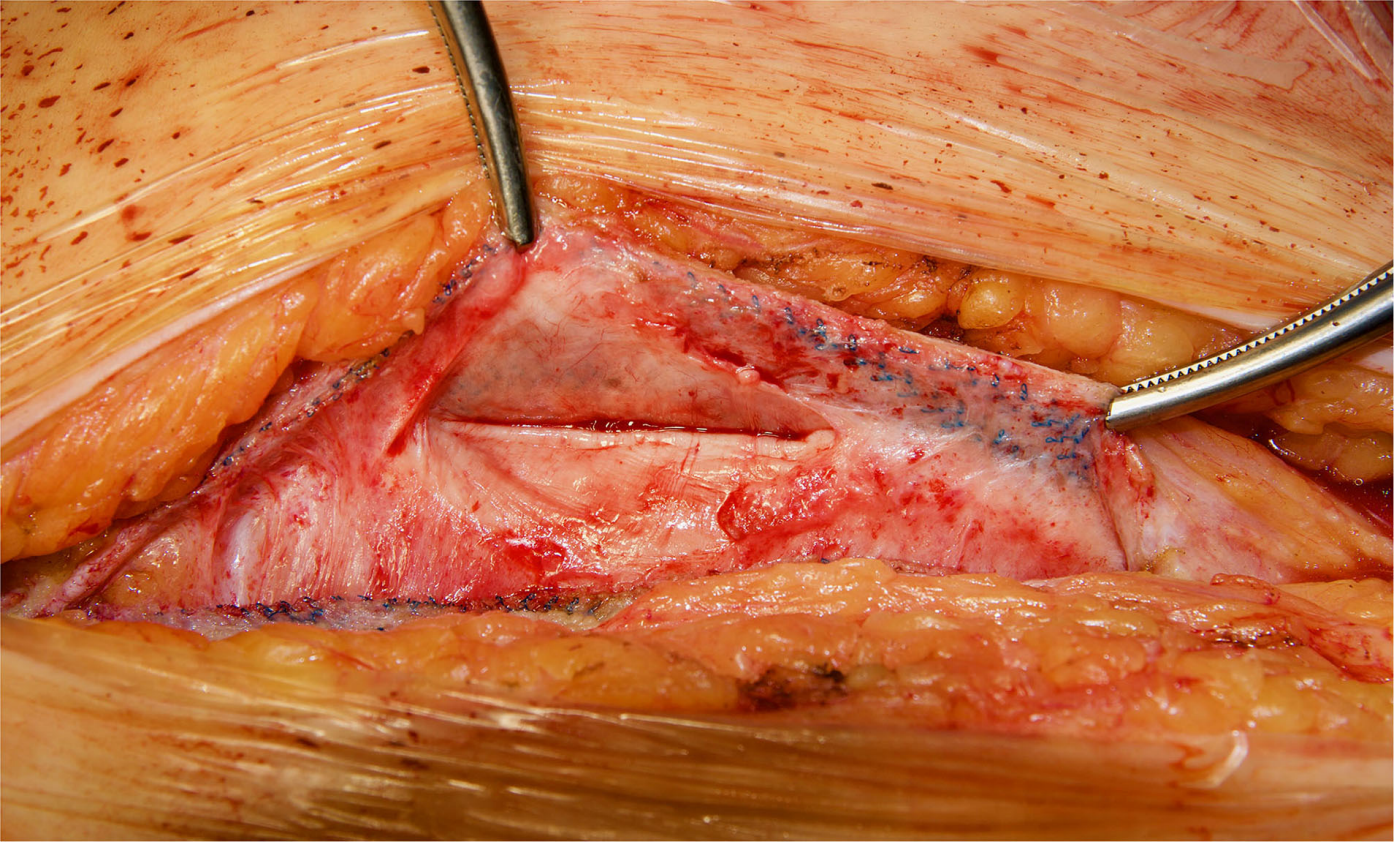
Reoperation through previous midline incision at 12 months. A dense fibrosis covering the peritoneum is observed.

**Figure 3:**
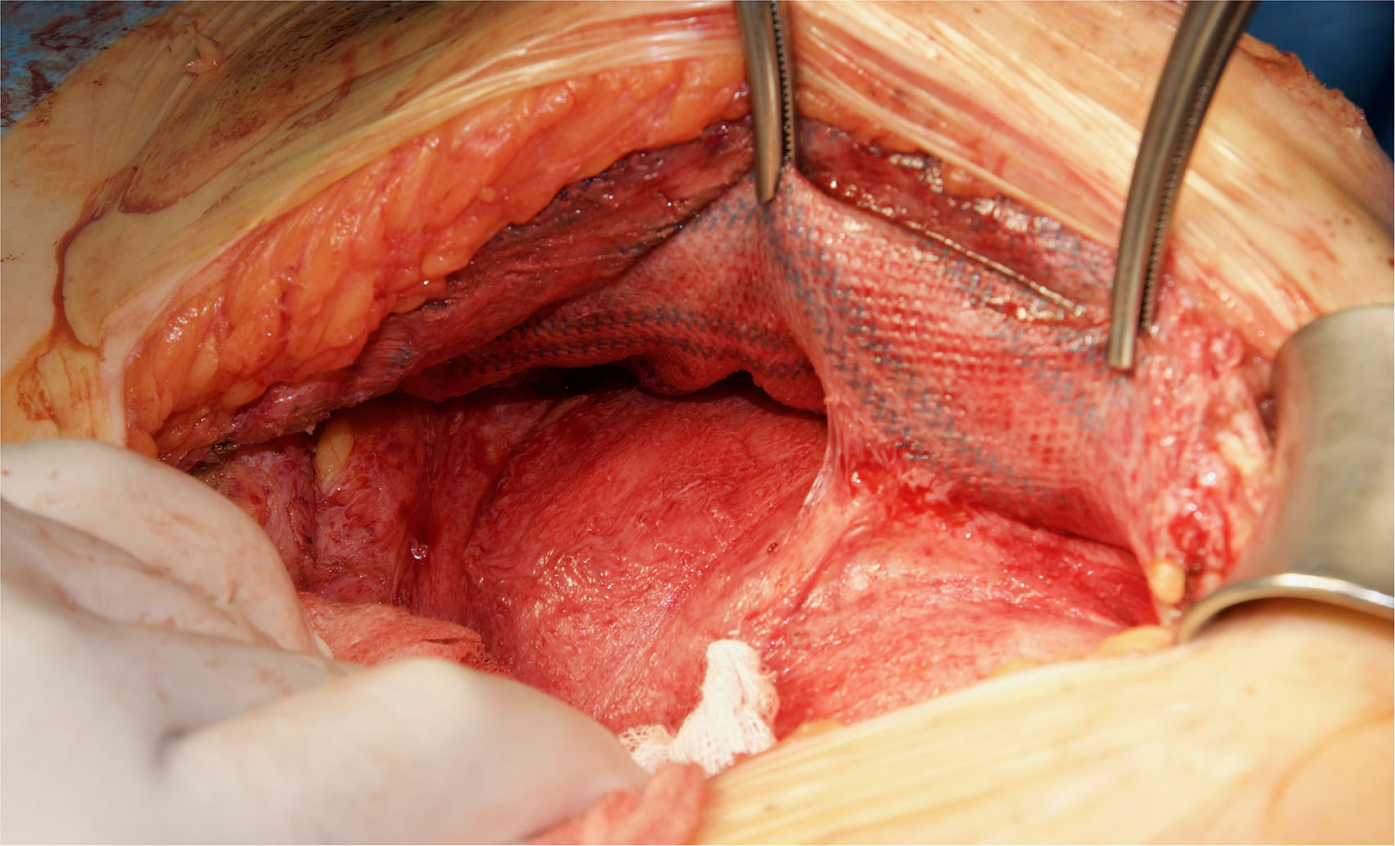
Redo retromuscular dissection in the case of Figure 2. Easy blunt dissection between peritoneum and permanent mesh could be achieved.

In the patients reoperated, we macroscopically observed a layer of fibrous tissue between the peritoneum and PM (Figure 1). This layer could be smoothly separated from the PM with blunt dissection (Figure 2). In fact, in four out of five patients with recurrence, the retromuscular preperitoneal plane could be dissected again without difficulty and used one more time for reconstruction (Figure 3).

**Figure 4:**
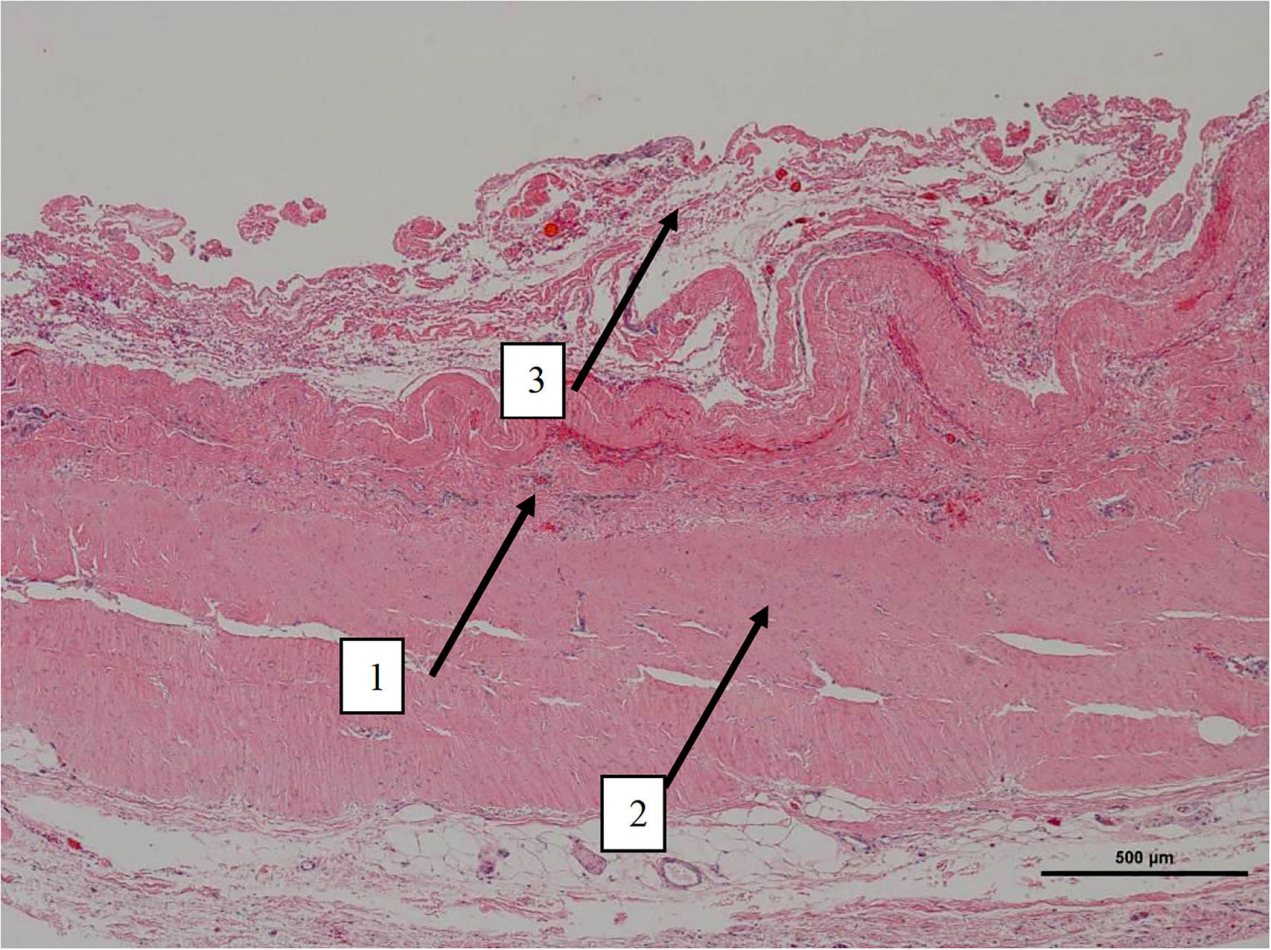
Low magnification picture of representative section on slide with hematoxylin & eosin staining. Arrow 1 denotes the layer where macrophages with intracytoplasmic particles are present as well as monofilament biomaterial. Arrow 2 represents an area with highly oriented and densely packed collagen. Arrow 3 on the periphery is composed of loosely arranged collagen fibers.

**Figure 5:**
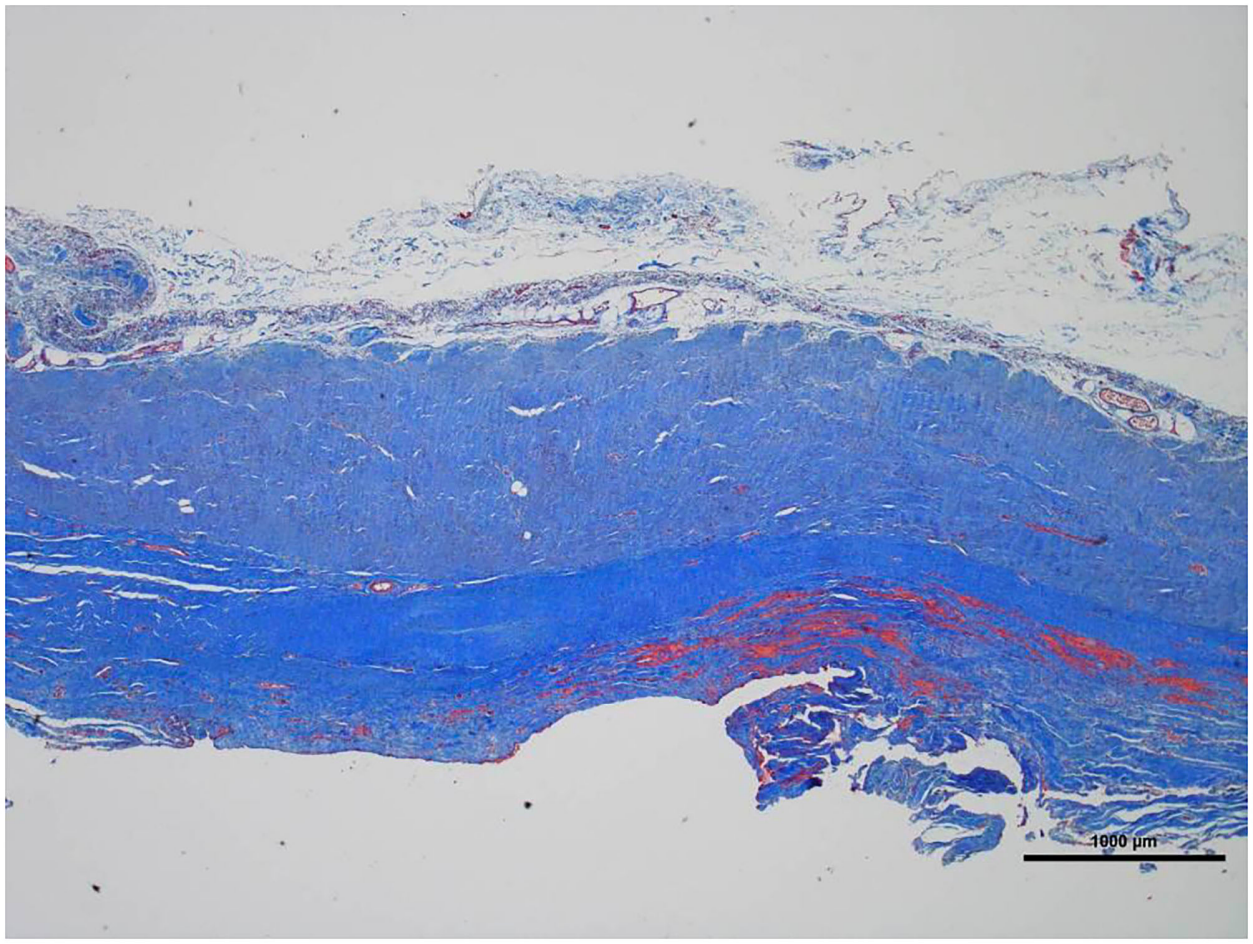
Low magnification picture of representative section on slide with Masson's trichrome stain. The connective tissue is composed almost entirely of collagen (blue staining tissue).

**Figure 6:**
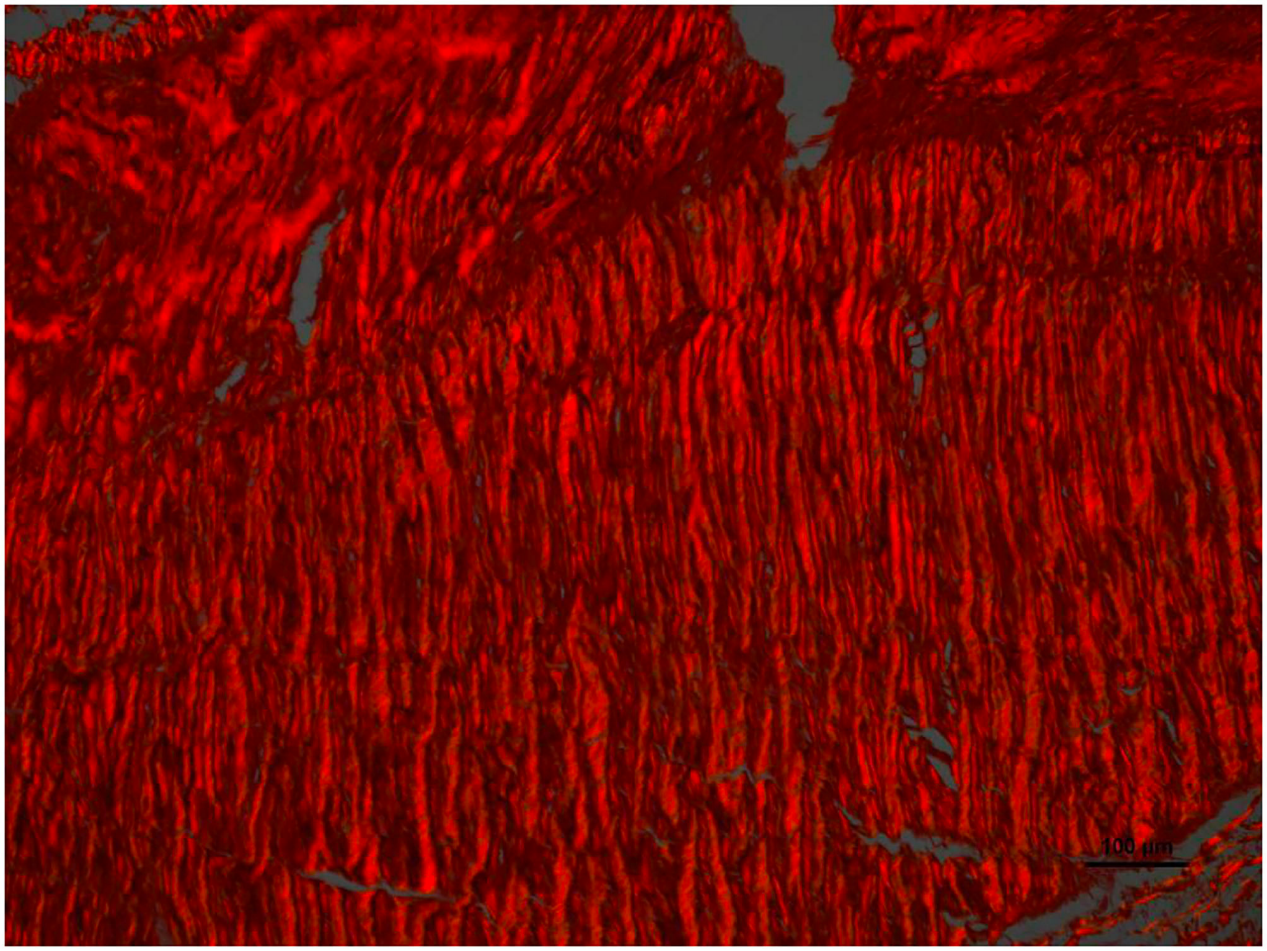
High magnification image of the area pointed with arrow number 2 in Figure 4. With Picrosirius red stain and polarized light microscopy the collagen is birefringent orange-red colored, highly oriented and densely packed.

**Figure 7:**
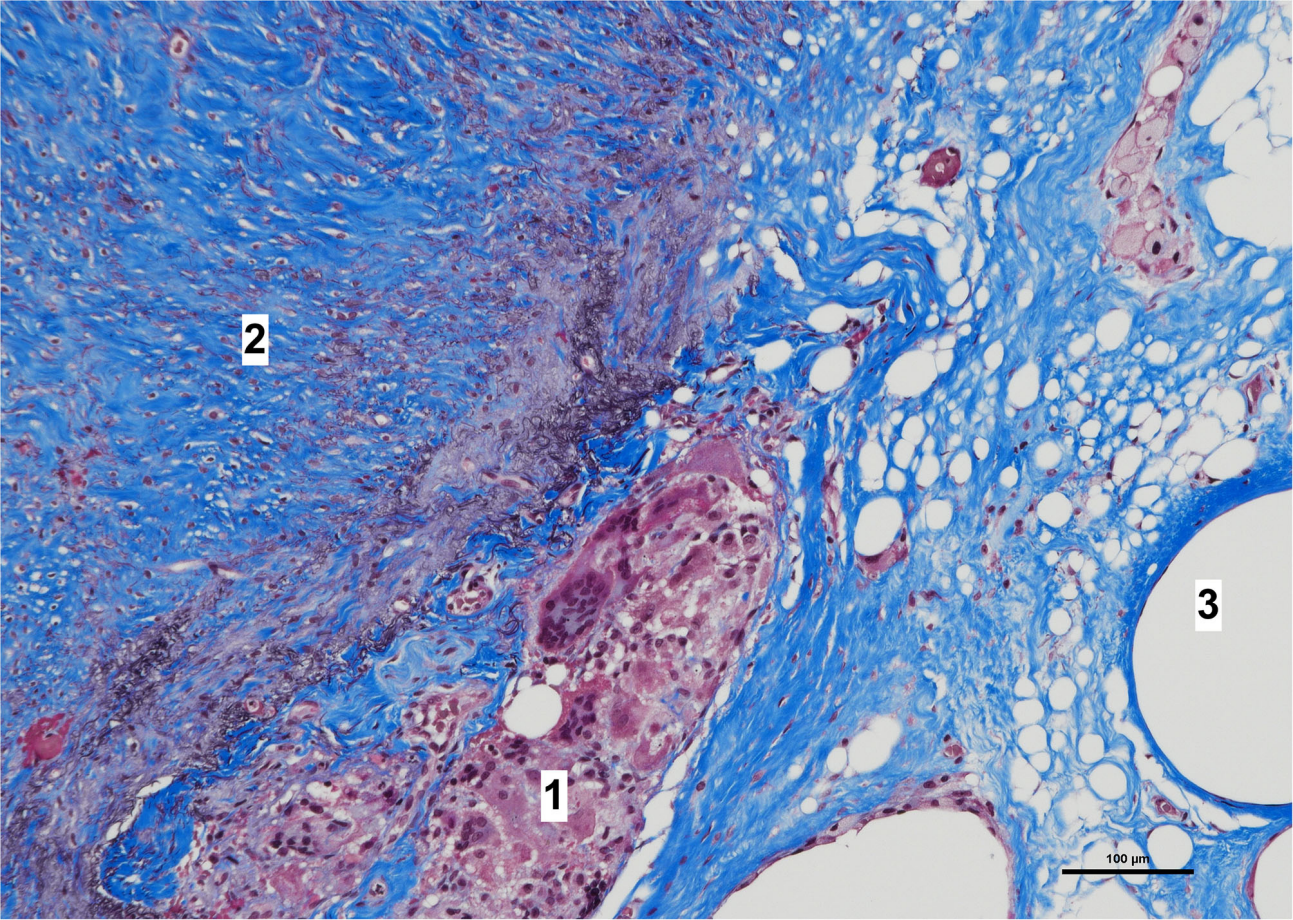
High magnification image of biopsy taken of case in Figure 1, with Masson's trichrome stain. Remnants of amorphous material (absorbable mesh) (1), between the fibrosis over the peritoneum, (2) and fibers of the permanent mesh (3).

Histologically, a thick fibrous capsule over the peritoneum was found in all specimens analyzed with hematoxylin-eosin stain (Figure 4). The identification of the fibrous tissue was confirmed in Masson's trichrome (Figure 5). Picrosirius red staining revealed that the composition consisted of mainly collagen scaffold (Figure 6). In the interesting case reoperated at 4 months due to intestinal obstruction for adhesion to the pelvic floor, an amount of amorphous material was still present with residual foreign body reaction among the fibrous layer, showing that the AM had only been partially integrated (Figure 7).

## Discussion

We started to use an AM along with a PM mainly to provide a barrier between intra-abdominal contents and PM in urologic cases. In these patients with previous cystectomies, where the peritoneum between both epigastric vessels is frequently removed, it was impossible for us to close the posterior layer despite the posterior component separation. We had the initial impression that the use of AM not only helped us to extend a very wide piece of PM without the need of transparietal fixations, but also that it could reinforce the posterior layer covering the inadvertent tears in the peritoneum. So, this became our model of AWR after posterior component separation. The satisfactory clinical results after using the combination of these meshes have already been published (5, 6, 12). Apart from the previous cystectomy cases, there are other circumstances that may expose the PM to the visceral content after a PCS (14). Peritoneal tears can be very common during a TAR procedure, meaning expert surgeons describe them as inevitable (15). The peritoneal layer is extremely thin and vulnerable to disruption between the midline preperitoneal fat and the anterior axillary line (5). The problem is not the tears that are seen and closed, but those unnoticed. Previous mesh implantations with or without infection, multi-recurrent hernias, AWR after open abdomen or resection of parietal tumors are other reasons that may prevent an adequate closure of the visceral sac or posterior layer. Acute internal hernias, that may occur in the immediate postoperative period, represent another complication after retromuscular hernia repair (16). All these problems can be solved by the placement of an AM between PM and peritoneum, creating an additional barrier facing intra-abdominal contents. The AM does not work like a conventional mesh and should be considered only as a tissue scaffold to reinforce the posterior layer and to cover inadvertent tears in the peritoneum. Based in our experience (5, 6, 12), we advocate to use the combination of meshes in complex abdominal wall reconstruction, particularly when there are concerns about appropriate closure of posterior layer. In our opinion, an incisional hernia that can be solved with a simple Rives-Stoppa procedure does not need this double mesh reconstruction.

We have then used this tissue scaffold, made of glycolic acid and trimethylene carbonate, that primarily degrades by hydrolysis and facilitates tissue generation and healing, as it has been observed in experimental settings (17, 18). These experimental findings have been confirmed in our clinical scenarios, as we have observed that AM is replaced by a fibrous tissue (Figures 6, 7). Apart from this clinical confirmation, we have also noticed that, when a new surgery in the abdominal wall was performed, a plane between PM and peritoneum could be dissected with blunt dissection (Figure 3). This maneuver is particularly important, as it may facilitate new retromuscular procedure to treat hernia recurrence or bulging. Masson's trichrome and Picrosirius red staining showed that a thick fibrous tissue had replaced the AM (Figures 6, 7). Rests of amorphous material still to be phagocyted, observed at 4 months, supports that the AM in combination with PM is, at least, partially responsible for this fibrosis (Figure 7). Here, it is important to remember that the histological analysis was made using the combination of AM and PM, and we do not know if the AM alone would generate the same response. Nonetheless, we do know, from our experience operating recurrences after retromuscular approach with PM, that dissecting a layer between peritoneum and previous retromuscular PM is almost impossible to achieve.

This is the first clinical report of macroscopic and histological analysis after the implantation of the combination of an AM and a PM. However, we are not the only ones that have used an AM as a barrier when performing retromuscular PM repairs. Liu et al. suggested, in an experimental model, that a physical barrier made of an AM may prevent adhesions to the PM (19). In this porcine model, self-made composite meshes made of non-absorbable and absorbable synthetic polyglactin materials were used in an intra-abdominal setting (intraperitoneal onlay). Although the use of an AM did not reduce adhesions to the PM, a thick fibrous capsule replaced the AM and therefore might represent an important layer to prevent intestinal erosion. The histological pictures shown in this experimental study are quite similar to those observed in our current study. Consequently, we propose that this important experimental analysis has a clinical correlation in surgical practice. Later, the same group published a multicenter experience using the AM as an interface between visceral contents and PM in cases where the peritoneum could not be completely restored (14). Probably looking for similar results, there are now several meshes that incorporate the combination of 3D absorbable or biological scaffold along with a PM that have been approved for clinical use in abdominal wall reinforcement by FDA (United States Food and Drug) administration: GORE^®^ SYNECORD^®^ Preperitoneal Biomaterial (WL Gore & Associates, Inc. Flagstaff, AZ, USA) and Ovitex^®^ Tissue Matrix (Tela Bio Inc. Malvern, PA, USA). The possibility of using the combination of 3D absorbable and permanent biomaterials might allow redoing the preperitoneal plane in case of reoperation for recurrence or bulging, but we have to wait for clinical results with these other biomaterials as the composition of them differs from the combination that we are currently using.

The configuration of the AM that we use is very different to the conventional macroporous woven knitted absorbable meshes (Vycril^®^ mesh) or to the more recent macroporous meshes with longer absorption times: Tigr^®^ Matrix or Phasix™. BIO-A^®^ Tissue Reinforcement is a 3D microscopic scaffold, 1.7 mm thick, that provides physical support to the extension of large PM, avoiding foldings, transparietal fixations and facilitating Stoppa and Taco configurations of PM at inguinal areas and posterior abdominal wall (12, 20). All these absorbable meshes are degraded by hydrolysis. Vycril^®^ is made of polyglycolic acid and polylactic acid; BIO-A^®^ Tissue Reinforcement, polyglycolic acid and trimethylene carbonate; Tigr^®^ Matrix, polyglycolic acid, polylactic acid and trimethylene carbonate; and Phasix™, poly-4-hydroxybutyrate. While complete resorption of Vycril^®^ mesh is at 2–3 months, BIO-A^®^ Tissue Reinforcement is around 6 months, Phasix™ 12–18 months and Tigr^®^ Matrix 3 years. Interestingly, we have observed some microscopic traces of the absorbable mesh at 1-year reoperations.

**Figure 8:**
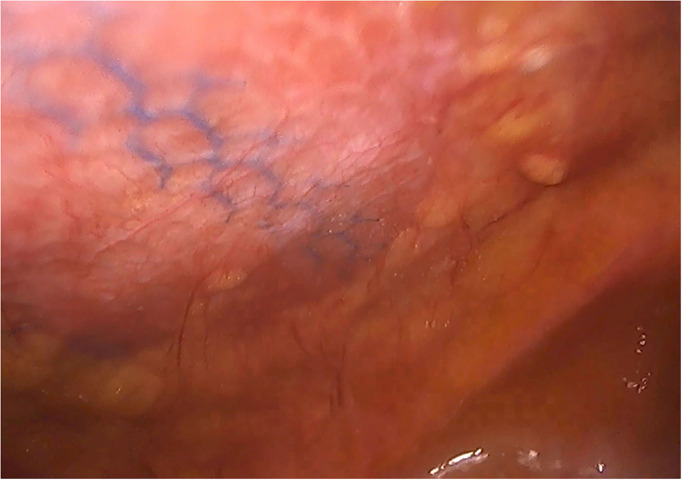
Laparoscopic reoperation after PCS technique with the combination of meshes.

**Figure 9:**
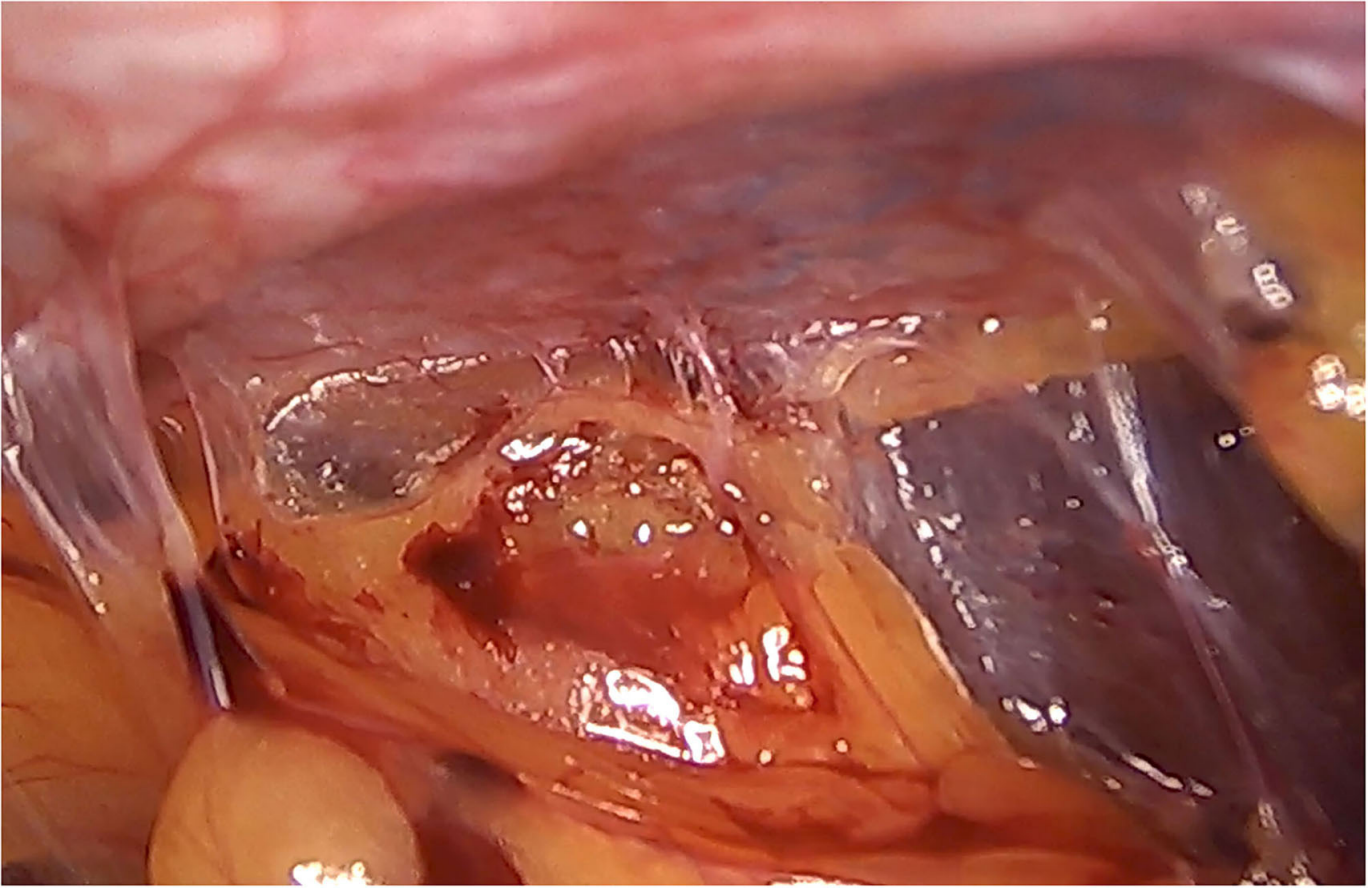
Intra-abdominal adhesions in another case of laparoscopic surgery after PCS technique with the combination of meshes. Tenacity score 2.

**Figure 10:**
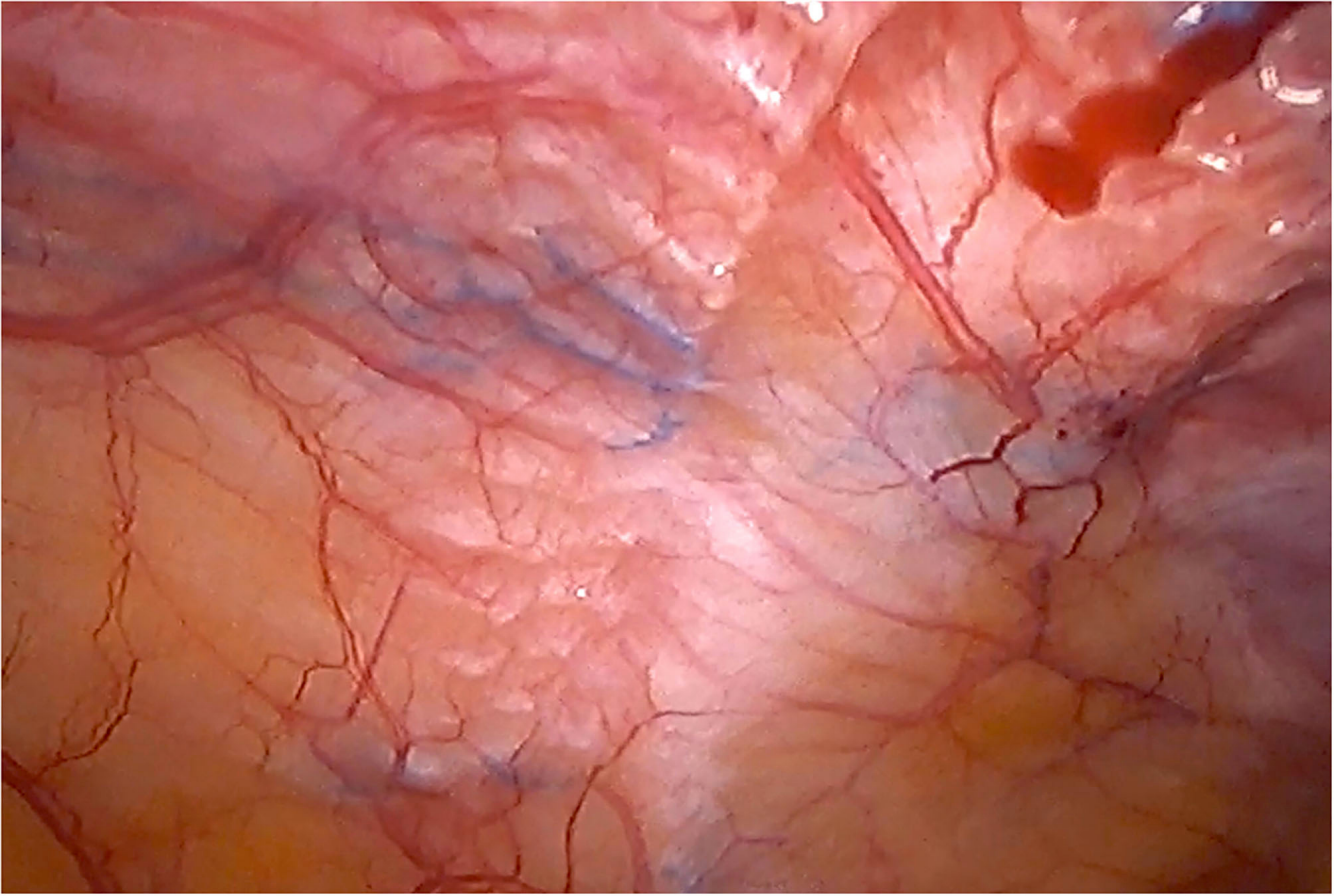
Laparoscopic reoperation after PCS technique with the combination of meshes. View of the edge of the permanent mesh without adhesions.

Although there are currently several adhesions classifications (21), we have chosen the classification that detail the tenacity of adhesion to previous mesh to better assess the reoperation findings (7, 8). As expected, we did not observe strong adhesions or viscera attached in any of the reoperated cases (Table 4). No adhesions (Figure 8) or filmy adhesions that only required blunt dissections (Figure 9) allowed four cases to be reoperated by laparoscopic approach despite the previous posterior component separation technique (Figure 10).

Our study has several significant limitations. Firstly, there is a lack of homogeneity in the causes that motivated a new surgical procedure. However, this study is focused on intraoperative macroscopic and microscopic features not related to its etiology. Secondly, no comparison that could provide useful information can be made among cases operated only using AM or PM. So that, no control group with only permanent mesh was analyzed. Nonetheless, these reoperations come from an international multicenter study that takes into account a large volume of patients with complex abdominal wall reconstruction using this technique.

## Conclusions

The observation of reoperated cases of abdominal wall reconstruction with the combination of an absorbable scaffold as an adjunct to permanent synthetic mesh represents an appropriate approach for complex incisional hernias as it may facilitate dissection between AM and PM granting new retro-muscular pre-peritoneal hernia redo in emergency or planned situations. We have witnessed that a fibrous protective layer rich in collagen fibers replaced the absorbable material. Few intra-peritoneal postoperative adhesions were observed, which were mainly filmy and easy to detach with blunt dissection, facilitating reoperations.

## Data Availability Statement

The raw data supporting the conclusions of this article will be made available by the authors, without undue reservation.

## Ethics Statement

The studies involving human participants were reviewed and approved by Comité Ético de la Universidad Francisco de Vitoria, proyecto 39/2019. The patients/participants provided their written informed consent to participate in this study.

## Author Contributions

MG-U, JL-M, AR, AM, and LB have contributed operating patients and writing draft of manuscript. JM-R and MP-F has elaborated tables and statistics. CS has elaborated figures, analyzed microscopic slides preparation, and review final version of manuscript. All authors contributed to the article and approved the submitted version.

## Conflict of Interest

MG-U, LB, JL-M, AR, and CS received honorarium for educational workshops organized by Gore. MG-U received speaker fees from Dipromed and B Braun. The remaining authors declare that the research was conducted in the absence of any commercial or financial relationships that could be construed as a potential conflict of interest.
